# Securing Educational Support for Children With Rare Genetic Conditions: Mothers' Experiences and Impacts on the Family

**DOI:** 10.1111/jir.70141

**Published:** 2026-07-08

**Authors:** Lowri O'Donovan, Anne‐Marie Burn, Tamsin Ford, Marianne van den Bree

**Affiliations:** ^1^ Centre for Neuropsychiatric Genetics and Genomics & Neuroscience and Mental Health Innovation Institute, Division of Psychological Medicine and Clinical Neurosciences Cardiff University Cardiff UK; ^2^ Department of Psychiatry University of Cambridge Cambridge UK

**Keywords:** educational support, qualitative, rare conditions, SEN

## Abstract

**Background:**

Children with rare genetic conditions are more likely to experience neurodevelopmental challenges requiring additional educational support. Although the governments in the United Kingdom and Ireland are committed to providing such support, securing it can be challenging for parents, with potential adverse implications for their mental health. Children who do not receive the educational support they need are at greater risk of poorer educational outcomes. This study aimed to remedy the lack of empirical data about the experiences of parents of children with rare genetic conditions in obtaining educational support and how these experiences affect them and their families.

**Method:**

Sixteen mothers were interviewed about their experiences of securing educational support for their child(ren) with a rare genetic condition. Qualitative data were collected during the Covid‐19 pandemic. Participants reflected on experiences both prior to and during this period. Data were analysed using Framework Analysis.

**Results:**

Five main themes were identified: (1) fighting for access into ‘the system’, (2) a lengthy process to secure support, (3) factors enabling access, (4) challenges after securing support and (5) impact of experience on mothers.

**Conclusions:**

Accessing educational support was challenging, lengthy and stressful, with negative effects on mothers' mental health and relationships with wider family members. Parents of children with rare genetic conditions may face additional challenges securing support.

## 1 Introduction

Rare conditions affect less than 1 in 2000 people (Department of Health and Social Care [DHSC] 2021). They have broad health impacts, requiring coordinated care from multiple specialist services, including education. For example, certain rare genetic conditions caused by Copy Number Variation (CNV; micro‐deletion or duplication of a chromosomal segment) are associated with neurodevelopmental conditions including autism spectrum disorder (ASD) (Chawner, Doherty, Anney, et al. 2021), attention deficit hyperactivity disorder (ADHD) (Niarchou et al. 2015), intellectual disability (Niarchou et al. 2014; D'Angelo et al. 2016), impaired motor coordination (Cunningham et al. 2019, 2021), emotional and behavioural difficulties (Cunningham et al. 2022) and sleep and neurological issues (Eaton et al. 2019; Cunningham et al. 2020; Agar et al. 2021; Chawner et al. 2023). Given the neurocognitive impacts of rare genetic conditions, educational support is an important aspect of their coordinated care (Walton et al. 2020).

Each of the UK nations and Ireland have signed up to the Convention on the Rights of Persons with Disabilities (United Nations 2006) and have outlined their commitment to inclusive education principles (Department for Education [DfE] 2025; Government of Ireland 2004; DfE, Department for Health [DfH] 2015; Scottish Government 2017; Welsh Government 2021). These commitments require education systems to ensure that all children, including those with rare genetic conditions, have equitable access to education and the opportunity to learn alongside their peers (United Nations 2006). Effective inclusion requires schools to make specific accommodations and provide appropriate support. Accordingly, children in the United Kingdom and Ireland who experience significantly greater difficulty in learning or in accessing school facilities compared with their peers should receive additional educational support (DfE 1998; Government of Ireland 2004; DfE 2015; Scottish Government 2017; Welsh Government 2021). Such support is dependent on individual need and can include in class support (e.g., allocated learning support and assistant to provide one to one support), physical and sensory support (e.g., movement breaks and access to sensory room within the school), or adaptations (e.g., exemptions from learning certain subjects; see Appendix 4). Where a child requires more support than their school can provide, an educational plan should be developed. This plan outlines the educational, health and social care provision that the local authority (LA) is legally obliged to deliver.

Although limited, evidence suggests that educational support can improve children's educational outcomes (Moore et al. 2018; John et al. 2022; Carroll et al. 2017); however parents in general report adverse experiences in obtaining educational support and children do not always receive the appropriate level of provision (Gains et al. 2025; Saxton, Burn, et al. 2025). These challenges were exacerbated during the Covid‐19 pandemic. In England, concerns were raised about increased delays in securing support, particularly following the UK Government's decision to relax the statutory timeframes LAs were required to meet when determining children's needs (DfE 2020). Parents also reported that children who already had educational support in place prior to the pandemic experienced its withdrawal (Alghrani and Byrne 2020; Couper‐Kenney and Riddell 2021; Baser et al. n.d.). This raises questions as to how inclusive education systems are in the United Kingdom and Ireland.

The process of obtaining educational support can also negatively impact parents' psychological health (Saxton, Burn, et al. 2025). Parents of children with rare genetic conditions may face additional challenges obtaining educational support for their child compared with parents of children with other underlying support needs. Parents who have the rare genetic condition can experience associated learning difficulties and may need additional support from professionals to navigate the system. There is also a lack of awareness of rare conditions among professionals (Wolstencroft et al., forthcoming) and their associated phenotypes are poorly understood, creating a significant barrier to the provision of appropriate educational support (Verger et al. 2020). Moreover, the presentation and associated care needs of children with rare conditions can be heterogeneous (McDonald‐McGinn et al. 2015), making it difficult to anticipate or plan for their specific needs. Evidence also suggests that school staff may not always take the needs of children with rare conditions seriously (Paz‐Lourido et al. 2020). Parents have reported that a rare genetic condition diagnosis can itself become a barrier to securing support in domains beyond education, as it can overshadow co‐occurring conditions and professionals can be reluctant to investigate additional support needs (Butter et al. 2024).

To our knowledge, the experiences of parents/carers seeking educational support for a child with a rare genetic condition, and the impact on them and their families remain undocumented. This project aimed to explore the experiences of parents/carers trying to secure educational support for their child with a rare genetic condition, and the impacts of these experiences on their lives, including their psychological health. Findings could help elucidate whether children with rare conditions in the United Kingdom and Ireland receive an inclusive education. Additional insights include whether parents may face additional challenges in securing support compared with parents of children with more commonly known conditions, which could help us begin to assess whether children with rare genetic conditions and their parents may represent a particularly underserved group.

## 2 Methods

### 2.1 Design

We followed the Consolidated Criteria for Reporting Qualitative Research (COREQ) checklist (Tong et al. 2007) to report our methods (Appendix 3). This project was approved by Cardiff University's School of Medicine's research ethics committee (project reference code: SMREC 21/47).

### 2.2 Participant Recruitment

We used purposive sampling to recruit parents of children with a diagnosed CNV or other rare genetic condition associated with developmental or physical health conditions. Both mothers and fathers were eligible to take part.

Participants were recruited via charities that support families affected by rare genetic conditions, such as Cerebra and Unique, and social media. We also emailed families who had participated in studies at Cardiff University (https://www.cardiff.ac.uk/centre‐neuropsychiatric‐genetics‐genomics/research/themes/developmental‐psychiatry/copy‐number‐variant‐research‐group; the ECHO [ExperienCes of people witH cOpy number variants] and IMAGINE‐ID [Intellectual Disability and Mental Health: Assessing the Genomic Impact on Neurodevelopment studies]) and consented to be contacted about future research opportunities. We hoped to recruit parents from a range of backgrounds, including sex, age, ethnicity and socioeconomic status (see Table 1 for additional characteristics) to increase the breadth of experiences represented; however, we did not actively target recruitment to certain groups and interviewed all eligible volunteers.

**TABLE 1:** Sample demographics.

	Total *N* (16)	%
**Mother characteristics**		
*Age (years)*		
< 20	0	0.0
20–29	1	6.3
30–39	3	18.8
40+	12	75.0
*Ethnicity*		
White	16	100.0
Minority ethnic	0	0.0
*Deprivation score*		
1st quintile	4	25.0
2nd quintile	3	18.8
3rd quintile	5	31.3
4th quintile	3	18.8
5th quintile	1	6.3

	Total *N* (19)	%
**Child characteristics**		
*Age (years)*		
Range	4–14	
Mean	9.4	
*Sex*		
Male	12	63.2
Female	7	36.8
*Ethnicity*		
White	17	89.5
Minority ethnic	2	10.5
*Inheritance status*		
Inherited	8	42.1
De novo	9	47.4
Missing	2	10.5
*Level of support*		
Mainstream school with educational plan^a^	6	31.6
Mainstream school without educational plan	7	36.8
Special school	6	31.6
*Stage of school*		
Nursery	1	5.3
Primary	13	68.4
Secondary	5	26.3
*Child likes school* ^b^		
Yes	14	73.7
No	5	26.3

*Note:* Inheritance status unavailable for two children.

^a^ Includes one child who attended a mainstream independent school.

^b^ As reported by mothers.

Interested parents emailed the lead researcher, who then phoned them to explain the study, confirm their child's eligibility and answer any questions they had. Interviews were scheduled over the phone, and the study information sheet and consent form were emailed to participants before their interview to allow time to read the documents.

### 2.3 Data Collection

An interview schedule was developed following consultation with a public and professional Advisory Group at Cardiff University (Appendix 1). Members of the Advisory Group (*n* = 8/23) who had a child with a genetic condition shared the challenges related to their child's schooling, which were then incorporated into the interview schedule.

Interviews took place between December 2020 and April 2021 during the Covid pandemic; however, participants were asked about their experiences in general, not those specifically during this time. Participants completed an online consent form via REDCap prior to the interview. All interviews were conducted online over Zoom by LOD and audio recorded. Interviews lasted about an hour. LOD was a PhD student when data were collected. She had previous experience and training in qualitative data collection.

At the start of the interview, participants were informed about the aim of the study and the main themes that would be covered in the interview. Participants were informed that they could skip questions, take breaks and ask for clarification at any point during the interview. First, demographic information was collected (see Appendix 1), which included families' socioeconomic status which was represented by a ‘deprivation score’. In the United Kingdom and Ireland, the relative deprivation of an area can be found using postcodes or residential area. These scores are calculated by the government of each respective nation based on several domains of deprivation, for example, income, employment, health and level of education. We used the quintile of deprivation associated with participants' home address to indicate families' deprivation score, with lower quintiles being indicative of higher levels of deprivation. We searched postcodes using the search tools associated with the 2019 English and Welsh indices of deprivation for participants residing in England and Wales and the 2017 indices for participants living in Northern Ireland. We searched residential areas within the 2022 indices for participants living in Ireland.

We also collected information on the inheritance status of children's genetic condition. Children were recorded as having an inherited condition if either biological parent also had the condition or as having a de novo condition if neither biological parent was known to have the condition.

The interview followed a semistructured format whereby follow up questions were asked to gather greater detail based on participants' answers. At the end of the interview, participants were given an opportunity to share additional information and were provided with a copy of their signed consent form and a study debrief form.

### 2.4 Analysis

All interviews were transcribed verbatim and entered into NVivo Version 13 for data management.

Transcripts were thematically analysed following the six stages of Framework Analysis (FA) (Ritchie and Spencer 2002). FA is a method which falls within the broader family of thematic analysis. The six stages include transcription, familiarisation, coding, development of an analytical framework, application of the analytical framework, charting data into the framework matrix, and data interpretation. We chose this approach because FA was developed for applied research and is particularly well suited to education research. It also provides a pragmatic, transparent and systematic process for identifying and interpreting themes, and facilitates the comparison of experiences between participants.

The analysis was primarily deductive, guided by the study aims. Two members of the research team LOD and AB independently read a subset of transcripts (*n* = 4) to familiarise themselves with the data and to begin identifying codes. They met to discuss the initial coding and developed an initial analytical framework. The analytical framework was then entered into NVivo, applied to the remaining transcripts, and iteratively refined in response to new data to ensure it captured all relevant data. See Appendix 2 for the final thematic framework.

Once all data had been coded, a framework matrix was exported from NVivo to Microsoft Excel. Charting involved summarising participants' experiences while retaining key verbatim quotes. Each cell in the matrix contained summarised data linked to specific codes and participant quotes that supported the findings. The matrix provided a detailed visual representation of the dataset, allowing LOD and AB to explore patterns and themes across both codes and individual participants and to finalise the themes.

## 3 Results

Sixteen mothers of a child with a diagnosed CNV or other rare genetic condition associated with developmental or physical health conditions were interviewed. No eligible fathers volunteered to take part. Three mothers had two children with a rare genetic condition, and therefore the information collected related to 19 children. Table 1 presents participant demographic information (e.g., children's mean age, sociodemographic information).

### 3.1 Themes

Five main themes were identified: (1) fighting for access into ‘the system’, (2) a lengthy process to secure support, (3) factors enabling access, (4) challenges after securing support and (5) impact of experience on mothers. Table 2 outlines the main themes and lists corresponding subthemes. The main themes are described below and illustrated with participant quotes below and in Table 3.

**TABLE 2:** Final themes and subthemes.

Theme	Fighting for access into ‘the system’	A lengthy process to secure support	Factors enabling access	Challenges after securing support	Impact of experience on mothers
Subthemes	(a) Rules and requirements	(a) Long waiting lists for public services	(a) Professional advocates	(a) The limitations of educational plans	(a) Increased stress and psychological adversity
	(b) Limited knowledge of rare genetic and neurodevelopmental conditions	(b) Local authority conduct	(b) The visibility of children's needs	(b) Future uncertainty	(b) Feeling a ‘problem parent’
	(c) Limited resources	(c) (Un)recognition and (un)acceptance of need	(c) Personal circumstances		(c) Familial relationships

**TABLE 3:** Main themes illustrated with participant quotations.

Main theme	Participant quotations
Fighting for access into ‘the system’	‘… the school kept saying, “well we cannot apply for the educational plan until we have got an official thing,” because although we'd got his diagnosis of [genetic condition], they did not think that would be sufficient enough to get the educational plan, so when we finally got that [ASD] diagnosis, it was a massive relief’. (M1)
	‘[child's paediatrician] put it in [child's] statement that [child] is probably going to need assessing for ADHD. I mentioned this to [child's] class teacher and TA … “oh well [child's] not ADHD, I know what ADHD is, [child's] definitely not got ADHD”. Within two weeks of being within special school and seeing the paediatrician [child] was medicated for ADHD … it just shows that they have no idea what it can look like’. (M3)
A lengthy process to secure support	‘… it went right up to the day before the court case, and then [the LA] turned round and conceded … the solicitor actually said that's what they do … “it will not go to court because I guarantee you like somewhere in that week before they will give you what you want” … they have no hope in hell of winning the case, they just keep it up to the very last minute’. (M7)
	‘… I feel like LAs sort of almost give lip service to it. “Oh, we'll invite the parents in for a way forward meeting,” and then nothing happens so the parents feel like they are getting somewhere and then they are just using it as another delaying tactic’. (M13)
Factors enabling access	‘where I am now, I probably would not be without the school support … literally fighting my corner every step of the way’. (M5)
	‘you just have to go on Facebook … There's so much stuff out there that parents do not realise’. (M8)
	‘it sounds bizarre but when COVID hit, [child] actually was able to go into school and because there was only about eight in the class [child] flourished in that time and developed so much more and like the teachers were like, “I cannot believe how much [child's] come along” … so they realised then just how much [child] actually needed that more one to one’. (M1)
Challenges after securing support	‘… unless [support listed in the educational plan is] quantified and specified, it's basically worth nothing’. (M8)
	‘I said, “you know, what about [child's plan] and [child's genetic condition]?” … and [the teacher] said, “what report?” and I said, “you never read the report?”’ (M9)
Impact of experience on mothers	‘I almost had an emotional breakdown, it was dreadful. At the time I was working full time … it was quite a stressful job as it was. I was coming home to emails from school, “oh this has happened, that's happened,” and then my grandma died as well so I was just‐ I'd got to the point where I just could not take anymore’. (M2)
	‘… if I cock this up, that's my child's education ruined, and it was such a pressure on us as parents that really made the decision to employ a solicitor because we just thought we could probably do this ourselves but we'll always kick ourselves if we do not employ a legal person … This is [child's] future we are talking about’. (M13)
	‘I did not even know I was going to be such a like, I dunno, stickler, keep on fighting for what I knew was right’. (M10)
	‘I feel like the pressure is ever so much on the woman … [my partner] will do all the laughy jokey stuff and the fun dad stuff … I deal with the serious stuff … the weight was on my shoulders’. (M8)
	‘… it affects obviously your relationship with your child cause you are trying to do paperwork and you are trying to sort out issues and they want to play and I mean like literally [child] would say, “can you come and play with me?” and I'd have say, “well no, mummy's got to do this.”’ (M13)

Responses to questions 4 and 5 on the interview schedule (‘What, if any, support does your child receive at school?’ and ‘What support does your child need at school?’) were not specifically related to the aims of the current study; however, because answers to these questions could be informative for readers regarding the support needs of children with rare genetic conditions, we have summarised mothers' responses in Appendix 4.

#### 3.1.1 Theme 1: Fighting for Access Into ‘the System’

Engaging with the additional educational support service—referred to as ‘the system’—was described as a ‘fight’ or ‘battle’ with many mothers encountering several barriers to accessing support. Some reported that certain rules and requirements, which did not align with official guidance, prevented access. For example, one mother was advised to pursue an additional ASD diagnosis because her child's genetic diagnosis would not be considered ‘sufficient enough’ by the local authority (LA) to qualify for an educational plan. Following confirmation of the ASD diagnosis, she was relieved to be able to secure an educational plan.

Another common challenge was the limited knowledge among professionals about rare genetic conditions. One mother was told her child's school was unable to provide support because staff did not understand the child's genetic condition but stated they would be better able to support if the child had a diagnosis of ADHD or ASD. Some mothers believed that professionals had outdated or ‘old fashioned’ stereotypes about neurodevelopmental conditions, for example, the belief that children with ASD cannot make eye contact. Some educational and LA professionals seemed confident in their own assessment of children's needs even when they were inaccurate, which sometimes led to the dismissal of parents' and other experts' opinions.

Mothers described their experience of fighting to access an underfunded system staffed by overworked professionals. Some reported that LAs intentionally withheld or misrepresented information about available support to ‘try and put people off’ applying and to protect resources. One mother, for example, was misinformed about the behaviours her child needed to display to qualify to attend an ASD specialist school, later discovering that the school had a cap on the number of students with educational support needs it could admit.

Mothers felt they were fighting for their child's basic ‘rights to an education’. Several expressed their child had been denied legally entitled support, which they described as a form of discrimination. One participant expressed that children with educational support needs are not given the support they need to achieve their potential and are denied the same opportunities given to their peers.

#### 3.1.2 Theme 2: A Lengthy Process to Secure Support

Mothers shared that it took several years to secure educational support, largely due to long waiting lists for public services. For example, one mother waited 2 years for her child's ASD diagnosis, which was necessary to progress her child's educational plan application. LAs frequently failed to follow statutory processes and time limits. Mothers sympathised with ‘completely overworked’ staff, linking delays to high levels of stress, staff absences and turnover, all of which further compromised the system. They also recognised that delays caused by the Covid pandemic were unavoidable.

Several families had taken their LA to tribunal to challenge decisions denying them support. However, tribunal waiting lists were described as extremely long. In some cases, LAs conceded just before the tribunal hearing, sometimes at the ‘very last minute’, which mothers perceived as a deliberate delaying tactic to postpone providing support.

LAs were also criticised for issuing ‘useless’ educational plans that required redrafting, and for holding meetings with parents that offered little more than ‘lip service’, seemingly intended to placate them until a later date.

The extent to which children's difficulties were outwardly visible impacted the time it took to obtain support. Several mothers referenced ‘masking’, where individuals conceal their challenges to fit in socially. For example, one mother shared that her child gets very upset during lessons because they cannot understand the work, but they mask this from the teacher, making their support needs less visible.

Two mothers shared that it took time for them to come to terms with their child's genetic condition which delayed the process of applying for support. One mother, who had two children with the genetic condition, reflected that she started the process later for her eldest child, as she needed to go through a process of acceptance first. Acceptance was also challenging when others, including professionals, told them their child had no underlying diagnosis and suggested their child's difficulties were ‘all in my head’. One mother even completed a college course in educational support needs to reassure herself she was not being misled by the school, which claimed her children did not need support.

#### 3.1.3 Theme 3: Factors Enabling Access

Mothers described the impact of having advocates in obtaining their child's educational support. One mother reported ‘things started to turn around a lot quicker’ after her child's paediatrician instructed the school about the support her child needed. However, this was not always the case as another mother, who was supported by her children's private tutor, shared that the school still did not ‘want to draw a conclusion and discuss anything at all’. One mother, who did not have an obvious advocate, went to great lengths to secure support, reaching out to the local Member of Parliament, the Child Commissioner and the National Autistic Society, who then assisted her.

Some parents benefited from strong peer support, helping each other to complete application forms. Online parent groups also existed to provide both practical and peer support. However, not all parents were aware of these groups.

During the Covid pandemic, some mothers said their children continued to attend school during lockdown, and this provided an opportunity for schools to see that their child needed additional support. Teachers were able to observe that children performed better academically and appeared happier learning in smaller sized classes.

Mothers reflected on personal circumstances and ‘slight perks’ that helped them secure support. These included financial resources to employ lawyers or commission private educational psychologists. Some also noted that their occupations facilitated access to relevant professionals and increased their knowledge about the educational support system. Acknowledging the challenges they faced securing support despite these advantages, mothers expressed concern for families without such resources and the potential long‐term consequences for their children, describing them as a ‘generation of children that just get lost’.

#### 3.1.4 Theme 4: Challenges After Securing Support

Mothers continued to face challenges even after formally securing support. The legally binding nature of educational plans reassured parents their child would receive the stipulated support. However, some mothers believed that schools and LAs were allowed the flexibility to interpret their content and provide less support if the educational plan was too vague.

Mothers also reported instances where teachers were uninformed about the content of their child's educational plan. In some cases, it was unclear whether the teacher had not read the plan or was even aware of its existence.

Some mothers noted their child's school was unable to provide the agreed support. One shared that her child's first school, a mainstream school, was the wrong setting and could not meet her child's needs. This was recognised by the school and the family. Another described having to amend her child's educational plan to secure a place at a special school; however, she had to go through the tribunal process in order to do this. A recurring theme was the limited knowledge that school staff had about how to support children with educational support needs, which hindered appropriate provision. One mother expressed frustration that her children's school relied on her for guidance on how to provide psychological support, despite her not being ‘an expert’ and ‘[I'm] just a mother’. Additionally, there were instances where schools falsely claimed support was being provided, for example, claiming they were helping with administering medication, and this led the parents to formally complain to the governor's board.

One mother reported that she had to continue to inform teachers about their non‐inclusive, judgemental attitudes and language. She speculated this was because of traditional approaches to education and insufficient teacher training on neurodiverse children's learning needs.

Mothers reflected that their child would likely need educational support throughout their education and that they had ‘years of this to come’. Indeed, one participant faced challenges securing a suitable college placement for her child nearing the end of secondary school. Ensuring the appropriate level of support was maintained during annual reviews was also difficult. The rarity of children's condition meant mothers were uncertain as to what future support their child might need. One mother shared that she and the school did not know what to expect from the child as time went on and that they would have to adapt and respond over time.

#### 3.1.5 Theme 5: Impact of Experience on Mothers

The demands on mothers applying for support were akin to a professional job, involving meetings, paperwork, emails and research, which caused ‘massive stress’. Some had left or changed their jobs, or reduced working hours, to cope. Mothers faced additional personal stressors, including challenges associated with their child being unsupported. The stress was intensified as mothers felt they were fighting for their child's education, and therefore, their future. The high stakes of not securing support compounded the pressure on mothers to succeed. Several mothers had been prescribed medication for associated stress, anxiety and low mood.

Mothers felt uncomfortable hearing what they felt were accusations about their child and being regarded as ‘a problem parent’ when disagreements arose between them and the school about their child's needs. However, the process of seeking support also revealed personal qualities they had not realised about themselves before, for example, patience and perseverance.

Some mothers reported that they were more heavily involved than their partner in securing educational support for their child and had therefore been more impacted by the process. Many mothers attended school meetings seemingly alone, and in cases where one parent had made professional adjustments, it was mothers who were reported to have done so. Mothers primarily spoke in first person singular (‘I’) versus first person plural (‘we’) or third person (‘he/she’ or ‘they’).

One mother thought her partner lacked the knowledge and confidence to address their child's educational needs. Her partner's employer was also less flexible in letting him attend school meetings, requiring him to take annual or unpaid leave, which was not financially viable. She believed her partner felt ‘a bit of an odd part’.

The unequal roles between mothers and fathers as reported here strained some parental relationships. One mother shared that it ‘nearly broke me and my partner up’. Another mother resented the impact the process had on her relationship with her child.

## 4 Discussion

This study aimed to investigate the experiences of parents/carers seeking to secure educational support for their child with a rare genetic condition, and the impact of these experiences on them and their families. Mothers' experiences were grouped into five main themes: fighting for access into ‘the system’, a lengthy process to secure support, factors enabling access, challenges after securing support and impact of experience on mothers. Our findings indicate that parents of children with a rare genetic condition struggle to access educational support, and that these experiences can negatively impact mothers' mental well‐being and family relationships. These findings are consistent with previous research exploring parents' experience of seeking support for special educational needs. Future research should explore whether mothers of children with a rare genetic condition face additional challenges in securing educational support compared with other parents of children who struggle in school, although this cannot be inferred from the current study.

Although the respective governments of the UK nations and Ireland have committed to provide inclusive education systems for all children (DfE 2025; Government of Ireland 2004; DfE, DfH 2015; Scottish Government 2017; Welsh Government 2021), our findings suggest that mothers of children with a rare genetic condition must overcome significant barriers before the necessary accommodations and support are implemented and maintained. Mothers who took part in this study believed that the need to fight for support amounted to discrimination against their child, raising serious questions about the effectiveness of inclusive education in these countries.

Mothers' experiences of the process were challenging and in line with previous research on parents' efforts to secure educational support in general (Gains et al. 2025; Saxton, Matthews, et al. 2025). It was striking that some mothers perceived local authorities employed ‘tactics’ that delayed the provision of resources, raising concerns about systemic practices that may deny children their right to an accessible education. This practice was thought to be in response to the limited resources available. Such action directly undermines the Convention on the Rights of Persons with Disabilities (United Nations 2006) and governments' commitment to inclusive education. Policymakers should review current guidance and legislation to close loopholes that allow such ‘tactics’ to persist.

These practices and mothers' sense of not being believed about their child's need for educational support contributed to fractious professional–parent relationships. Improved collaboration between parents and professionals can benefit such relationships and lessen the advocacy burden on mothers (Ryan and Quinlan 2018). Collaborative working could also be particularly beneficial for understanding the support needs of children with rare genetic conditions. This is particularly important given that existing literature on the educational needs of children with rare conditions is sparse (Röjvik et al. 2024), and that difficulties experienced by this group can be modified subtly, for example, neurodevelopmental conditions in children with CNVs (Niarchou et al. 2015). Indeed, our findings pointed toward insufficient expertise in neurodevelopmental conditions in schools, and knowledge of rare genetic conditions was even more limited. Interestingly, our study indicated that educators and LA professionals appeared confident in their assessment that children did not need educational support. These findings are in line with reports that while parents believe educational and LA professionals receive insufficient training in identifying additional educational support needs in children, professionals themselves report they receive adequate training (Gains et al. 2025). Improved teacher training may, in part, lead to improvements in educators' knowledge of such conditions (Nash et al. 2016; Atkinson et al. 2025), and may also go some way to improving parent–professional relationships and inclusivity. However, as has been recommended previously (Gains et al. 2025), future research should also explore the effectiveness of current training.

School staff cannot be expected to have in depth knowledge about all rare conditions and their associated needs. Parents can accumulate valuable knowledge about their child's needs from the various specialists they see, their own research and their personal experience of being a parent and can therefore provide helpful insights to assist professionals. Their knowledge should be seen as an asset to facilitating inclusion and a resource for education staff. Beyond parental expertise, resources have been created for educators to help them support children with specific rare genetic conditions, or rare conditions more generally. For example, Reilly and Stedman (2013) provide a detailed overview of the educational support needs of children with 22q11.2 deletion syndrome. Röjvik et al. (2024) argue that children with rare conditions require a holistic approach to education that incorporates both their medical and educational needs and recognises the way in which these mutually influence each other. They have created a five‐step approach to help educators put in place such support. An online resource (https://www.findteacherresources.co.uk/) has also been developed by researchers at the University of Surrey and the Cerebra Network for Neurodevelopmental Disorders to support educators working with children who have genetic syndromes.

While parents struggle to access educational support for their child in general (Gains et al. 2025; Saxton, Matthews, et al. 2025), the lack of professional knowledge of rare conditions described by mothers here could suggest that parents of children with a rare genetic condition may face additional barriers to securing support. We did find some supporting evidence of this; for example, one mother was advised to secure additional, more commonly known diagnoses such as ASD because a child's genetic condition was thought insufficient to grant them an educational plan.

Mothers' reports suggested that families' socioeconomic background could influence their ability to secure support. For example, parents who pursued their cases at tribunal courts after their application for educational support was unsuccessful perceived that having financial resources to employ a legal representative was advantageous for success. These experiences support evidence for inequities in the educational support system (Campbell 2023), and strengthen reports that children with a rare genetic condition from more deprived backgrounds are particularly vulnerable (Lee et al. 2024).

The combined challenges of caring for a child with multiple needs as well as obtaining the educational support they need may render mothers of children with rare genetic conditions particularly vulnerable for adverse mental health consequences. For example, not only do these mothers have to navigate the educational support system, but when they are advised to seek additional diagnoses for their child, as noted above, they also have to steer additional overstretched public services, such as child and adolescent mental health services (British Medical Association 2019), which can also increase parental stress levels (Crane et al. 2016). This is supported by accumulating evidence that mothers of children with rare genetic conditions experience higher rates of mental illness compared with parents/carers in the general population and parents/carers of children with other disabilities (Baker et al. 2021; Fitzgerald and Gallagher 2022; Niarchou et al. 2022). Parents may wish to seek additional diagnoses; however, it should not be required when a child's rare condition and presentation already evidence need for support. This could not only save parents significant stress but also reduce the demands on children's diagnostic services, which have seen a sharp increase in referrals since the pandemic (Huang and Ougrin 2021).

There was a clear sense from mothers taking part in this study that their motivation was to secure their child's educational rights and future opportunities. Their child was evidently their priority and therefore measures aiming to reduce mothers' stress but which do not meet their child's underlying educational needs could likely be ineffective, especially given the bidirectional relationship between parent stress and child behavioural problems (Woodman et al. 2015). Intervention strategies need to adopt effective family‐centred care that addresses the whole family's needs (Walton et al. 2020).

Lastly, our findings reflect reports that mothers of children with a rare condition are typically more involved in their child's care compared with fathers (Eurordis 2017). Increased caregiving burden for mothers of children with educational support needs was particularly marked over the Covid‐19 pandemic (Baser et al. n.d.), and some of the associated problems noted by mothers in this study may have heightened over this time, for example, increased strain on marital relationships. One mother noted her partner's employer lacked flexibility in allowing him to take time off work and his limited confidence in advocating for their child as contributing factors. Efforts could be made by support services and employers to enable both parents to be more involved in their child's care, which could help empower fathers and alleviate pressure from mothers.

## 5 Limitations

Although conducting interviews online increased geographical reach and representation of recruitment, this study reflects mothers' experiences only as no fathers volunteered to participate. Additionally, the demographic makeup of our sample lacks diversity. For example, those who took part were primarily of white ethnic background, middle aged and recruited from previous research studies conducted by the team. While gathering a deep understanding of this group's experiences is important, we recognise the likely biases of our sample and that our findings may only speak to a narrow range of people. We cannot assume that the experiences of the mothers taking part in this study reflect the experiences and perspectives of other parents and therefore the transferability of our findings to other groups is limited. Increased targeted recruitment could have enabled us to explore a broader range of perspectives, including those of fathers, children and teachers.

Our investigation provides insights into parents' difficulties securing educational support. However, the needs of children with rare conditions are varied (Chawner, Watson, and Owen 2021), and parents must access and coordinate multiple public services. Given the constraints on public services more generally, parents likely experience high stress levels when liaising with these other services. Investigating their experiences beyond education would provide a fuller picture of their experiences and the impacts of securing support more generally, helping policy makers and public service leaders better understand how to improve experiences for parents and mitigate harmful effects.

This study was concerned with parents' experiences in general and not those specifically over Covid‐19; however, given our interviews were conducted over the pandemic, we recognise this context will have influenced participants' experiences. Indeed, Covid was mentioned by several parents as a factor that both positively and negatively impacted their child's access to support. A post‐pandemic follow up study could be valuable to investigate whether mothers' perspectives have changed. Finally, more detailed information about the children, for example, their developmental stage would be informative.

## 6 Conclusion

This is the first known qualitative investigation into the experiences of mothers seeking educational support for children with rare genetic conditions and the impact of such experiences. While additional studies are needed to clarify whether the experiences described are reflective of other parents, our study supports reports that the educational support system is stressful to navigate, under‐resourced and negatively impacts parent/carer mental health (Gains et al. 2025; Saxton, Burn, et al. 2025; Saxton, Matthews, et al. 2025). Ultimately, these challenges are likely to have detrimental consequences for both children's educational outcomes and parents' wellbeing. We also observed that navigating the educational support system can negatively impact wider family life.

While our findings suggest children with rare conditions may face marked challenges in accessing educational support, we recognise that many of our findings reflect the experiences of parents navigating the educational support system in general. Similarly, many of the recommendations in this paper are not new and would serve all parents and children navigating the educational support system.

## Funding

This work was supported by the Knowledge Transfer Partnership project Improving Mental Health for Children With Neurodevelopmental Disabilities between Cerebra and Cardiff University (KTP) (KTP: 11189) and by Innovate UK and Llywodraeth Cymru.

## Conflicts of Interest

The authors declare no conflicts of interest.

## Data Availability

The data that support the findings of this study are available from the corresponding author upon reasonable request and upon agreement by funders.

## Appendix 1 Parent Interview Schedule

Begin each interview explaining the study, its background and its aims and question topics.

Introductory questions

1. Collect demographic data about the parent *(their age, sex, ethnicity, and postcode)*
2. Collect demographic data about the child *(their age, sex, ethnicity, their genetic condition, is the genetic condition inherited or de novo)*
3. School related information:
  - What school year is your child in?
  - What type of school does your child go to *(special or mainstream)*?
  - Do they have an EHCP/educational plan?
  - Overall, do you think your child likes school?

Theme 1: Your child in school

1 How do you think your child generally feels when they are in school?
2 What do you think makes them feel this way?
3 What does your child like about school?
4 What does your child dislike about school?

Theme 2: Support at school

4 What, if any, support does your child receive at school?
5 What support does your child need at school?
6 (If relevant): What has your experience been like to obtain support for your child at school?
7 How has the level of support your child receives impacted them?

Theme 3: Relationships

8 How does your child get along with other children at school?
9 Has your child got a best friend or close circle of friends?
10 Has your child experienced bullying?
11 How does your child get along with the teachers at their school?
12 How has your child's relationships with others at school impacted them?

Theme 4: Impact of experience

11 How has your child's experience of school impacted them?
12 For example, at home, academically, socially, emotionally
13 How has your child's experience of school impacted you and the rest of your family?

## Appendix 2 Final Thematic Framework

1. **Obtaining support** (this is an overarching title to help organise the codes below. Codes below refer to parents' experiences of securing support at school for their children).
  1. Motivation: What motivates parents to secure support for their children; how parents' motivation can compare to the motivations of services.
  2. Barriers and facilitators: Barriers and facilitators commonly experienced by parents when obtaining educational support.
  3. Interaction with professionals: Parent–professional interactions as reported by parents.
  4. Positive experiences: Examples of positive experiences of obtaining support and praise for particular members of staff.
  5. Parent narrative: The process of obtaining educational support as told by parents (specific descriptors, emotions, etc.).
2. **Impact of support** (this is an overarching title to help organise the codes below. Codes below refer to the range of impacts that the level of educational support received by children can have).
  1. Impact on children: Immediate and long‐term impact of support received at school on children both outside of and at school.
  2. Impact on parents: The impact the level of educational support has on the lives of parents.
  3. Remaining issues: What educational support has not been able to achieve (e.g., change in teacher attitudes/understanding).
  4. Wider impacts: The impacts of educational support that go beyond the child and family (e.g., other pupils).

## Appendix 3 COREQ (Consolidated Criteria for Reporting Qualitative Research) Checklist

A checklist of items that should be included in reports of qualitative research. You must report the page number in your manuscript where you consider each of the items listed in this checklist. If you have not included this information, either revise your manuscript accordingly before submitting or note N/A.

**Table:**

Topic	Item no.	Guide questions/description	Reported on page no.
Domain 1: Research team and reflexivity			
*Personal characteristics*			
Interviewer/facilitator	1	Which author/s conducted the interview or focus group?	4
Credentials	2	What were the researcher's credentials? For example, PhD and MD	4
Occupation	3	What was their occupation at the time of the study?	4
Gender	4	Was the researcher male or female?	4
Experience and training	5	What experience or training did the researcher have?	4
*Relationship with participants*			
Relationship established	6	Was a relationship established prior to study commencement?	4
Participant knowledge of the interviewer	7	What did the participants know about the researcher? For example, personal goals and reasons for doing the research	4
Interviewer characteristics	8	What characteristics were reported about the interviewer/facilitator? For example, bias, assumptions, reasons and interests in the research topic	N/A
Domain 2: Study design			
*Theoretical framework*			
Methodological orientation and Theory	9	What methodological orientation was stated to underpin the study? For example, grounded theory, discourse analysis, ethnography, phenomenology and content analysis	5
*Participant selection*			
Sampling	10	How were participants selected? For example, purposive, convenience, consecutive and snowball	3
Method of approach	11	How were participants approached? For example, face‐to‐face, telephone, mail and email	3–4
Sample size	12	How many participants were in the study?	5
Non‐participation	13	How many people refused to participate or dropped out? Reasons?	N/A
*Setting*			
Setting of data collection	14	Where was the data collected? For example, home, clinic and workplace	4
Presence of nonparticipants	15	Was anyone else present besides the participants and researchers?	N/A
Description of sample	16	What are the important characteristics of the sample? For example, demographic data and date	Table 1
*Data collection*			
Interview guide	17	Were questions, prompts, guides provided by the authors? Was it pilot tested?	Appendix 1
Repeat interviews	18	Were repeat interviews carried out? If yes, how many?	N/A
Audio/visual recording	19	Did the research use audio or visual recording to collect the data?	4
Field notes	20	Were field notes made during and/or after the interview or focus group?	N/A
Duration	21	What was the duration of the interviews or focus group?	4
Data saturation	22	Was data saturation discussed?	N/A
Transcripts returned	23	Were transcripts returned to participants for comment and/or	N/A

Topic	Item no.	Guide questions/description	Reported on page no.
		Correction?	
Domain 3: Analysis and findings			
*Data analysis*			
Number of data coders	24	How many data coders coded the data?	2
Description of the coding tree	25	Did authors provide a description of the coding tree?	Final codes in Table 2
Derivation of themes	26	Were themes identified in advance or derived from the data?	5
Software	27	What software, if applicable, was used to manage the data?	5
Participant checking	28	Did participants provide feedback on the findings?	N/A
*Reporting*			
Quotations presented	29	Were participant quotations presented to illustrate the themes/findings? Was each quotation identified? For example, participant number	Table 3
Data and findings consistent	30	Was there consistency between the data presented and the findings?	Yes
Clarity of major themes	31	Were major themes clearly presented in the findings?	Results Section
Clarity of minor themes	32	Is there a description of diverse cases or discussion of minor themes?	Results Section

*Note:* Developed from Tong A, Sainsbury P, Craig J. Consolidated criteria for reporting qualitative research (COREQ): a 32‐item checklist for interviews and focus groups. *International Journal for Quality in Health Care*. 2007. Volume 19, Number 6: pp. 349–357.

## Appendix 4 Summary of Mothers' Responses to the Questions: ‘What, If Any, Support Does Your Child Receive at School?’, and ‘What Support Does Your Child Need at School?’

**Table:**

Participant	Type of school attended by child	What, if any, support does your child receive at school?	What support does your child need at school?
M1	Mainstream	Educational plan; one to one support for 50% of the school week; emotional therapy/support; play therapy.	Increased one to one support (however mother appreciates that it is beneficial for child to have some independent learning time).
M2	Mainstream	Extra time for exams; therapy room in school; SENCO monitors mental health by distributing ‘a regular’ mental health questionnaire to pupils.	None.
M3	Special	Educational plan; sensory support.	None.
M4	Special	Educational plan; smaller class sizes; music therapy; speech and language therapy.	None.
M5	Mainstream	Educational plan; one to one support for 4 h a day; emotional literacy sessions; movement breaks; occupational therapy support equipment (for example, a writing slope).	None.
M6	Mainstream × 2	Both children: differentiated learning; pastoral care; limited speech and language therapy.	Educational plan; subject specific support in mathematics; additional speech and language therapy.
M7	Mainstream	Educational plan; one to one support.	None.
M8	Mainstream	Educational plan; learns in a class with other children with educational support needs; teaching assistant support; sensory room in school; emotional literacy sessions; pastoral support.	More independent learning without the teaching assistant to support independence; more support with academic skills.
M9	Mainstream	English literacy classes; exemption from certain subjects (foreign languages); smaller class size for certain subjects; movement breaks; a social club for children with educational support needs.	Educational plan; further exemptions from certain classes; a reader and scribe for exams; life skills support.
M10	Special	Educational plan; speech and language; occupational therapy; vision therapy.	None.
M11	Special	Educational plan; smaller learning classes; movement breaks; soft playroom in school; sensory room in school; classes are formed based on ability not children's age.	None.
M12	Mainstream	Life skills support (cooking, cleaning, counting money); speech and language therapy; teaching assistant support; smaller learning classes for English and mathematics.	Wider practical support; behavioural support; sensory support; one to one support.
M13	Mainstream independent	Educational plan; one to one support; smaller class size; occupational therapy; speech and language therapy.	Better understanding of neurodiversity by school staff.
M14	Mainstream × 2		Child 1 is supposed to get pastoral support; physiotherapy; and the school should be helping to administer medication, but parent does not see evidence of any support being provided. Mother would like more teaching assistant support for both children; an educational plan for both children; and uniform adaptations for Child 2 to support their sensory needs.
M15	Special × 2	Both children: educational plan; nurse onsite; smaller class sizes; sensory barn at school; therapeutic dog; Child 1: speech and language therapy; life skills support; Child 2: one to one support.	Child 1: None; Child 2: too young to know fully what support they need yet.
M16	Mainstream	Educational plan; teaching assistant support; speech and language therapy; small group work; occupational therapy; physiotherapy.	One to one support; mother does not see evidence of the support agreed within the educational plan being implemented.

*Note:* Information presented summarises information provided by mothers from memory during interviews and may not represent an exhaustive list of all support stipulated in children's educational plan or provided by the school.

Abbreviation: SENCO, Special Educational Needs Coordinator.
